# A cognitive remediation training for young adults with psychotic disorders to support their participation in education — study protocol for a pilot randomized controlled trial

**DOI:** 10.1186/s40814-020-00579-0

**Published:** 2020-04-27

**Authors:** Lana K. M. Otto, Jacomijn Hofstra, Michelle G. Mullen, Derek Malenczak, Nynke Boonstra, Lisette van der Meer, Wim Veling, Cees Boerhout, Gerard D. van Rijsbergen, Jos de Vries, Boudien van der Pol, Gerdina H. M. Pijnenborg, Lies Korevaar

**Affiliations:** 1 Research and Innovation Center for Rehabilitation, Hanze University of Applied Sciences, Zernikeplein 23, 9747 AS Groningen, The Netherlands; 2 Faculty of Behavioural and Social Sciences, Department of Clinical Psychology & Experimental Psychopathology, University of Groningen, Grote Kruisstraat 2/1, 9712 TS Groningen, The Netherlands; 3 Transitions to Adulthood Center for Research, Department of Psychiatry, University of Massachusetts Medical School, Worcester, MA 01545 USA; 4 Department of Psychiatric Rehabilitation and Counseling Professions, School of Health Professions, Rutgers University, 675 Hoes Lane West, Piscataway, NJ 08854 USA; 5 Department of Research and Education, Friesland Mental Health Services, PO Box 932, 8901 BS Leeuwarden, The Netherlands; 6 NHL Stenden University of Applied Science, Research group Care & Welfare, Rengerslaan 8, 8917 DD Leeuwarden, The Netherlands; 7 Department of Rehabilitation, Lentis Psychiatric Institute, E 035, 9471 KA, Zuidlaren, The Netherlands; 8 Rob Giel Research Center, University Medical Center Groningen, PO Box 30001, 9700 RB Groningen, The Netherlands; 9 Faculty of Behavioural and Social Sciences, Department of Clinical and Developmental Neuropsychology, University of Groningen, Grote Kruisstraat 2/1, 9712 TS Groningen, The Netherlands; 10 University Center for Psychiatry, University Medical Center Groningen, University of Groningen, PO Box 30001, 9700 RB Groningen, The Netherlands; 11 GGz Drenthe Mental Health Institution, Dennenweg 9, 9404 LA Assen, The Netherlands

**Keywords:** Cognitive remediation, Education, Psychotic disorders, School performance, Young adults

## Abstract

**Background:**

Most severe mental disorders have their onset between the age of 17 and 27, a time when many young adults begin participating in secondary or post-secondary education. The cognitive deficits typically associated with psychiatric disorders, especially psychotic disorders, increase the risk of leaving school early, which can lead to a reduction in employment opportunities later on in life and, in turn, a poorer long-term prognosis. Therefore, specific interventions aiming to improve these cognitive functions are needed. Cognitive remediation (CR) aims to improve cognitive functioning and may increase real-world functioning in educational performance. This study aims to examine the feasibility and applicability of a CR training named Mindset for students with psychotic disorders in the Netherlands.

**Methods/design:**

Sixty students diagnosed with a psychotic disorder and currently reporting cognitive deficits will be included from four Dutch Mental Health Care institutes. Half of the participants (*N* = 30) will be randomly assigned to the CR training consisting of twelve, individual, weekly 1-h meetings. The other half will be assigned to an active control condition consisting of twelve weekly assignments that will be sent by email aiming to improve school performance. Students will be evaluated at baseline (T0), directly after finishing the CR training or control intervention (T1), and 6 months later (T2). Treatment feasibility will be the primary outcome, using evaluation forms, interviews with trainers and participants, number of study drop outs, and patient eligibility and recruitment rates. School functioning, cognitive functioning, and strategy use will also be assessed to get a preliminary idea of the potential effectiveness of the intervention.

**Discussion:**

The CR training in this study will provide real-world examples and exercises aimed to teach useful strategies to cope with the cognitive deficits experienced by students with psychotic disorders. Furthermore, since students with other psychiatric disorders might also experience cognitive deficits, the results of this study may also provide some further implications for future studies on the effect of this CR training for students with these disorders.

**Trial registration:**

The study was registered with Trialregister.nl, no. NL6590 (NTR6764), date registered: September 7, 2017. Register name: Mindset. A cognitive rehabilitation training for young adults with psychotic spectrum disorder in an educational setting: A pilot study.

Protocol version: 3, date December 23, 2019

## Background

Most psychiatric disorders include having cognitive problems and have their onset between the age of 17 and 27 [1], which is often the period in which many young adults participate in higher education.

Cognitive deficits are negatively associated with functional outcomes like social functioning, independent living skills, occupational functioning, and also level of educational success [2–4].

Between 6 and 20% of the students participating in higher education experience mental health problems [5–8], and nearly 55% of this group is severely hindered by their psychiatric disorders during college [9]. Research shows that students with psychiatric problems spend on average more time on their education per week. However, they also receive lower grades compared to students without psychiatric problems [10] and are at higher risk of early school leaving [10, 11], which results in having reduced chances of obtaining employment afterwards. This reduction in social participation often leads to worsening of the prognosis of the disorder on a long-term basis [12]. Offering support to these students while they are enrolled in school might increase their chances of educational success [13] and could mitigate the issues of unemployment and social isolation seen in the older adult population. The cognitive deficits associated with psychiatric disorders are, compared to other psychiatric disorders [14], most prominent in people with psychotic disorders [15, 16]. These cognitive deficits are often already present before the first onset of the psychotic disorder [17].

One intervention aimed at decreasing cognitive deficits is cognitive remediation (CR). CR is defined as “a behavioral, training-based intervention that aims to improve cognitive processes (attention, memory, executive function, social cognition, or metacognition) with the goal of durability and generalization” [18]. Previous studies have shown that CR has positive effects on almost all cognitive abilities of people with schizophrenia [13, 19–22], with considerable variability in effects between studies. Various moderators for the effectiveness of CR were identified. Previous studies, for example, have demonstrated that baseline cognition can positively impact the outcome of CR, with severe cognitive impairment at baseline resulting in poorer study outcome post-training compared to moderate cognitive impairments [23]. In addition, medication usage [24], gender [25], age of onset [26], and self-confidence [27] have been identified as moderating factors. In addition, the use of “bridging activities” to daily life, like supported employment, has been shown to be more effective than cognitive training alone [28].

Most studies on the effects of CR have focused either on cognition [29, 30] or cognitive functioning in an employment context [19–21, 31–34] in people with schizophrenia. These studies indicated that CR improves cognitive functioning, especially when combined with supported employment [19–21, 35].

Positive results have been associated with young adults; specifically, young adults have more cognitive improvements after CR [36–38], which could be possibly explained by brain plasticity in young adults early in the course of the illness [39, 40]. Offering CR to young adults might therefore lead to better results as compared to older adults with more chronic forms of psychotic disorders [39, 40]. The few studies that have focused on CR for adolescents or young adults with psychotic disorders in an educational setting [13, 41, 42] demonstrated a positive effect on cognition [42], academic functioning, and self-esteem [13], as well as a decrease in self-reported academic difficulties [41].

A CR for young students with a psychotic disorder is not available in the Netherlands yet. This study therefore aims to examine the feasibility and applicability of a CR training in an educational setting for students with psychotic disorders. To do this, a CR training developed by Mullen and colleagues [41] was translated to Dutch, adapted to the Dutch context, and was named Mindset. Mindset will be studied as a pilot randomized controlled trial at four mental health care (MHC) services in the Netherlands.

## Methods

### Participants

Sixty students will be recruited from four Dutch Mental Health Care services (in Dutch: Geestelijke Gezondheidszorg; GGz), namely GGz Drenthe, GGz Friesland, Lentis Groningen, and the University Center of Psychiatry (UCP), Groningen. The recruitment will be done by MH professionals, who will distribute information brochures at the participating MHC institutions. Inclusion criteria are (i) receiving treatment from an Early Intervention Psychosis team of one of the participating MHC services; (ii) officially diagnosed with a psychotic disorder by one of the participating MHC services; (iii) self-report of cognitive deficits which interfere with the ability to participate in coursework; (iv) aged above 18; (v) participating in mainstream postsecondary education: intermediate vocational education, higher vocational education, or university; (vi) at least 1 year remaining in education after start of the training or control intervention, and (vii) able to give written informed consent. Exclusion criteria are (i) having participated in a supported education or cognitive remediation program in the past year and (ii) having an estimated IQ < 75 (identified by a MH professional). Daily treatment of the participants will be unchanged.

### Sample size calculation

Sample size calculations are not applicable since the study focuses on the feasibility and applicability of the cognitive remediation training. According to the literature, a sample size of 25 to 30 per group should be sufficient to estimate moderate effect sizes in a pilot test for adequacy and acceptability of the instruments using *α* = 0.5 [43, 44]. We therefore aim for 30 participants per group. Participants will be recruited from the four participating sites providing variation in demographic and clinical characteristics useful for conclusions on general feasibility.

### Study procedures

The Standard Protocol Items: Recommendations for Interventional Trials (SPIRIT) 2013 checklist [45] was adhered to and is provided in Additional file 1. After study procedures have been fully explained, participants will provide written informed consent and will then be randomized into either the CR training or the active control group (see SPIRIT 2013 Fig. 1).

**Fig. 1:**
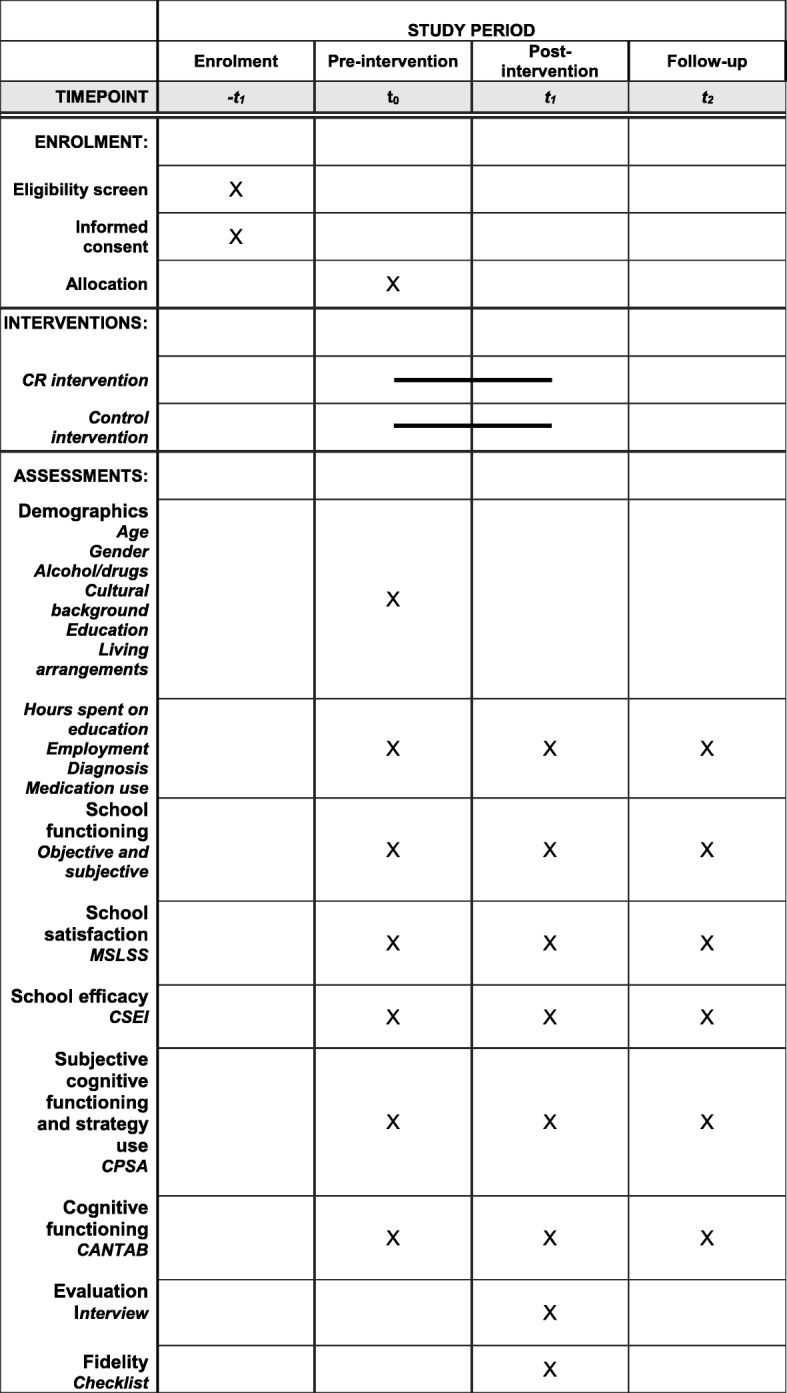
SPIRIT flow diagram of the study procedure showing enrolment, interventions, and assessments

Feasibility, applicability, and exploratory efficacy outcomes will be assessed at baseline (T0), upon completion of the CR training or control intervention (post-treatment; T1), and 6 months after T1 (follow-up; T2). The written evaluation forms for fidelity and feasibility will be completed by the trainers after each session. Evaluation interviews will be held at the end of the training (T1) with both participants and trainers and will be performed by the researcher involved.

Half of the participants will receive the CR training (intervention group), whereas the other half will receive study assignments without additional support (control group). Participants in the control group are offered Mindset 1 year after the experimental group completes the intervention. Participants will receive compensation (three times gift coupons of 12.50 euro each) for filling in the questionnaires at baseline (T0), post-treatment (T1), and during the follow-up, 6 months after T1 (T2).

### Interventions

#### CR training

The Mindset training is designed to improve the cognitive abilities of students with psychiatric disorders, including psychotic disorders, and is aimed at improving academic outcomes. The training consists of twelve individual, 1-h meetings spread over 12 weeks. Personal educational goals of the participants will be formulated during an introductory meeting (session zero). The students will be asked to relate the skills and strategies practiced during the training to their own educational goals. During the training, four cognitive domains will be targeted: (1) prospective memory, (2) attention and concentration, (3) verbal learning and memory, and (4) cognitive flexibility and problem-solving. All sessions follow the same structure: review of last session’s home exercises, overview and importance of the current session’s topic, practice exercises, and review of new home exercises. The training includes practical strategies such as agenda usage and paying attention during class, forming a direct link to educational settings. The trainers are professionals from the participating MHC services and received a 4-day training in the CR by the developer of the original CR training.

#### Control group

Mindset will be compared to an active control condition to control for attention. The control group also begins with a session zero to formulate their educational goals and will receive twelve weekly assignments via e-mail, without any face-to-face contact involved. The home assignments will take about 1 h to complete. No feedback will be provided on these assignments. The topics of the assignments are designed in such a way that they do not focus on cognitive functions but still provide some educational support. Examples of the exercises are “requesting for support,” “dealing with stress,” and “responding to feedback.”

### Measurements

A full list of measurements and measure points is given in the SPIRIT flow diagram (Fig. 1).

At baseline, the following demographic information will be collected per participant: age, gender, alcohol and drug usage, cultural background, education (previous and current), level of education, number of hours spent on their education per week (i.e., school credit hours and homework), living arrangements (living with parents/roommate(s)/partner and/or having children), employment information (number of hours), diagnosis (including age of onset and symptom severity), and medication use (name and frequency).

#### Feasibility, applicability, and fidelity outcome measures

The main study outcomes are study feasibility and applicability and will be measured using an evaluation form completed by the trainers, an interview with trainers and participants, and by examining study dropouts. In addition, patient eligibility and recruitment rates will be included as a measurement for feasibility and applicability of the training within this population as the number of potentially eligible people compared to the number of actual participants.

Training fidelity will be measured by the previously described interview and a session evaluation form, which contains a short checklist of all session components. The evaluation forms are designed in such a way that the trainer can provide information on how much of the sessions they have covered per exercise. The form also allows the trainer to provide feedback for each exercise within the session, as well as general feedback about the session. The fidelity measurements will provide greater understanding and description of how the training criteria have been attained, or why they may not have been attained.

The evaluation interviews will be semi-structured, audio-recorded (with consent), and transcribed. During the interview, questions like “Which session did you find most useful?” and “Are there aspects of the training that can be, or need to be, improved?” will be asked. All interviews will be encoded by two independent researchers. The codes obtained from both researchers will be compared and discussed, resulting in one coding file for each of the interviews.

#### Exploratory outcome measures

School- and cognitive functioning will be measured as exploratory outcomes to get a first impression of the efficacy of Mindset. To do this, objective and subjective school functioning, school satisfaction, school self-efficacy, subjective school functioning, strategy use, and cognitive functioning will be collected. In addition, information concerning interruptions in schooling (like school dropouts) will be gathered during the interviews.

#### Objective school functioning

School performance will be calculated as percentage of courses successfully completed using the following formula: $\frac{\mathrm{succesfully}\ \mathrm{completed}\ \mathrm{courses}}{\mathrm{number}\ \mathrm{of}\ \mathrm{courses}}\times 100\%$, because the number of courses will not be the same for all participants. Therefore, an absolute number will not provide enough information about the total performance per student.

#### Subjective school functioning

The Educational Barriers Questionnaire or EBQ (Mullen MG. Educational Barriers Questionnaire. 2016. Unpublished) will be used to measure self-reported school functioning. The EBQ is a questionnaire including 34 problems students might experience when going to school, such as problems with “taking notes” and “prioritizing tasks.” The EBQ uses a 5-point scale, ranging from 1 (not at all) to 5 (very often). Total scores on the questionnaire will be used for analyses, where higher scores indicate experiencing more problems.

#### School satisfaction

School satisfaction will be measured using the Multidimensional Students’ Life Satisfaction Scale (MSLSS)—school subscale [46]. This subscale consists of nine statements about school satisfaction, for example, “I look forward to going to school” and “There are many things about school I don’t like.” Respondents are asked to indicate on a 4-point scale ranging from 1 (rarely) to 4 (always) to what extent the statements are applicable to them. Negative items like “I wish I didn’t have to go to school” will be reverse-scored. Higher scores indicate higher levels of school satisfaction.

#### School efficacy

School efficacy will be measured using the College Self-Efficacy Inventory or CSEI [47]. The CSEI is a 22-item self-reported questionnaire measuring student confidence levels related to their use of post-secondary educational skills such as “write a course paper” and “ask a professor or instructor a question outside of class.” The CSEI uses a 9-point scale, ranging from 0 (totally unconfident) to 8 (totally confident). Total scores will be used for analyses, where higher scores indicate higher confidence levels throughout the scale.

#### Strategy use and cognitive functioning

Strategy use and subjective cognitive functioning will be measured using the Cognitive Problems and Strategies Assessment, or CPSA [32]. This questionnaire contains 35 cognitive problems, such as “I have difficulty remembering to do things that I have scheduled” and 30 strategies like “I keep a written list of things I need to do.” The CPSA uses a 4-point scale ranging from 0 (rarely/never) to 3 (always). Total scores on “cognitive problems” and on “strategy use” will be calculated and used for analysis separately. Higher scores on “cognitive problems” indicate having more problems, and higher scores on “strategy use” indicate using more strategies.

#### Cognitive functioning

Objective cognitive functioning will be measured using the Cambridge Neuropsychological Test Automated Battery or CANTAB [48]. The CANTAB includes seven different modules measuring various neurocognitive functions and processes, including working memory, episodic memory, executive function, emotion recognition, cognitive flexibility, processing speed, and sustained attention.

### Randomization and blinding procedures

Randomization will be done in blocks of four and six participants, stratified for gender to control for possible gender effects [25]. Participants will be randomly assigned to either the CR or control condition by an independent researcher. Assessments will be done by trained bachelor or master level students in Psychology or Nursing, supervised by senior psychologists. The assessors are blinded to the conditions of the participants.

### Handling and storage of data and documents

Data will be handled confidentially in compliance with the Dutch Personal Data Protection Act (*Wet Bescherming Persoonsgegevens*; WBP). Research assistants will assign each participant with a unique identification code. The key to this code and a subject identification list will be safeguarded by the project manager. All data will be stored and locked at the Hanze University of Applied Sciences Groningen. Raw research data will be stored until 15 years after finishing the study and will be destroyed thereafter according to the code of proper use (“Gedragscode Gezondheidsonderzoek”; www.fmwv.nl).

### Dissemination

Dissemination of the results of the project will take place through scientific publication(s) in (inter)national peer-reviewed journals; presentations and workshops during national and international conferences of associations of educational staff, mental health workers, and rehabilitation specialists; a closing symposium; online introductory course on Supported Education for mental health professionals, students, and educational professionals; newsletters; and social media (LinkedIn groups, Twitter, Facebook, etc.).

### Proposed analyses

Analyses will be performed according to the “intention to treat” (ITT) principle. Descriptive statistics will be provided for all normally distributed variables in the form of mean scores and standard deviations for each of the assessment instruments. Descriptive statistics of variables that are not normally distributed will be represented as median scores and ranges. Data from the interviews will be qualitatively analyzed. Differences in the quantitative outcomes will be examined for T0 to T2 using repeated measures of variance with the MANOVA test using 95% confidence levels providing estimates of potential treatment effects since real significance and hypothesis testing is not applicable for a pilot study.

## Discussion

Cognitive remediation (CR) has shown positive effects across almost all cognitive domains [13, 19–21]. However, it should be noted that most of the previous CR research was conducted among samples of older adults with mean ages that ranged between the 30 and 47 years [19, 22] and were focusing on cognitive functioning in an employment context [19–21, 31–34]. Most cognitive skills needed for employment are also necessary for educational success. Furthermore, successfully completing one’s education influences one’s future perspective on employment and consequently on one’s social inclusion. Decreased social inclusion can lead to a poorer prognosis [12], warranting for interventions like CR in an educational setting. However, CR in educational settings is still studied by only a few [13, 41, 42].

In addition, young adults in the early phase of illness may have greater brain plasticity, which is needed for recovery and learning. Offering CR to students aged between 17 and 27 might therefore lead to better results as compared to older adults with a more long-term psychotic disorder [39, 40].

This study will test the feasibility and applicability of a CR training named Mindset for students with psychotic problems in the Netherlands. Mindset incorporates practical strategies such as agenda usage and paying attention during class, forming a direct link to educational settings. The use of these “bridging activities” has been shown to be more effective than cognitive training [28].

Although this is a pilot study with a relatively small sample size, the results of this study will provide a good basis for further research on CR in the context of education. Furthermore, because cognitive deficits are not only seen in students with psychotic disorders, but also in students with other psychiatric disorders [2, 3, 35], the results of this study might also provide some implications for further studies on the effect of CR on students with other psychiatric disorders.

## Supplementary information

**Additional file 1.** SPIRIT 2013 Checklist.

**Additional file 2.** CONSORT checklist of information to include when reporting a pilot trial.

## Data Availability

Data sharing is not applicable to this article as no datasets were generated or analyzed during the current study.

## Abbreviations

CANTAB: Cambridge Neuropsychological Test Automated Battery
CCMO: Central Committee on Research Involving Human Subjects; in Dutch: *Centrale Commissie Mensgebonden Onderzoek*
CPSA: Cognitive Problems and Strategies Assessment
CR: Cognitive remediation
CSEI: College Self-Efficacy Inventory
EBQ: Educational Barrier Questionnaire
GGz: Mental Health Care institution (in Dutch: *Geestelijke Gezondheidszorg*)
HBO: Higher vocational education (in Dutch: *Hoger beroepsonderwijs*)
IC: Informed consent
MBO: Intermediate vocational education (in Dutch: *Middelbaar beroepsonderwijs*)
METc: Medical Research Ethics committee (MREC; in Dutch: *Medisch Ethische Toetsings commissie*)
MHC: Mental health care
MSLSS: Multidimensional Students’ Life Satisfaction Scale
SPIRIT: Standard Protocol Items: Recommendations for Interventional Trials
US: United States
WBP: Personal Data Protection Act (in Dutch: *Wet Bescherming Persoonsgegevens*)
WMO: Medical Research Involving Human Subjects Act (in Dutch: *Wet Medisch-wetenschappelijk Onderzoek met Mensen*)
WO: University education (in Dutch: *Wetenschappelijk Onderwijs*)
